# Siglec-15 on macrophages suppress the immune microenvironment in patients with PD-L1 negative non-metastasis lung adenocarcinoma

**DOI:** 10.1038/s41417-023-00713-z

**Published:** 2023-12-11

**Authors:** Ziqi Huang, Yan Guo, Baihui Li, Meng Shen, Yeran Yi, Li Li, Xiaohe Zhao, Lili Yang

**Affiliations:** 1 Department of Immunology, Tianjin Medical University Cancer Institute & Hospital, National Clinical Research Center for Cancer, Tianjin’s Clinical Research Center for Cancer, Key Laboratory of Cancer Immunology and Biotherapy, Tianjin, China; 2 Department of Good Clinical Practice Center, Shanxi Province Cancer Hospital/Shanxi Hospital Affiliated to Cancer Hospital, Chinese Academy of Medical Sciences/Cancer Hospital Affiliated to Shanxi Medical University, Taiyuan, China; 3 Department of Esophageal Cancer, Tianjin Medical University Cancer Institute & Hospital, National Clinical Research Center for Cancer, Tianjin’s Clinical Research Center for Cancer, Key Laboratory of Cancer Prevention and Therapy, Tianjin, China

**Keywords:** Tumour immunology, Cancer microenvironment, Immunosuppression

## Abstract

Sialic acid-binding immunoglobulin-like lectin 15 (Siglec-15) is an immune checkpoint molecule with sequence homology to programmed cell death ligand 1 (PD-L1), which is mainly expressed on macrophages and tumor cells. However, whether Siglec-15-induced immunosuppression and poor prognosis are independent of PD-L1 remains unclear. In this study, we collected samples of 135 non-small cell lung cancers and found that Siglec-15 and PD-L1 expression were independent in non-small cell lung cancer by multiple immunofluorescence staining. Siglec-15 on macrophages (Mφ-Siglec-15) was significantly associated with DFS (*p* < 0.05) in PD-L1^−^ patients with non-metastasis lung adenocarcinoma, not in PD-L1^+^ or lung squamous cell carcinoma patients. Moreover, stromal Siglec-15^+^ macrophages of Mφ-Siglec-15^+^PD-L1^−^ patients were significantly more than those of Mφ-Siglec-15^−^PD-L1^−^ patients (*p* = 0.002). We further found that Siglec-15^+^ macrophages polarized toward M2 and produced more IL-10, negatively associated with inflamed immunophenotype in PD-L1^−^ patients and may inhibit CD8^+^T cells infiltration. In conclusion, PD-L1-independent Siglec-15^+^ macrophages contribute to the formation of an immunosuppressive microenvironment in non-metastasis lung adenocarcinoma patients, which may cause a higher risk of recurrence. Siglec-15 could be a potential target for normalizing cancer immunotherapy, benefiting patients who fail to respond to anti-PD-L1 therapy.

## Introduction

In the past decade, the first-line treatment for patients has been completely changed, and this shift can be attributed to the discovery of the programmed cell death receptor 1 (PD-1) immune escape signaling pathway [1, 2]. Lung cancer remains the leading cause of cancer deaths [3, 4]. The use of immune checkpoint inhibitors (ICIs) has brought great benefits to a subset of NSCLC patients, enabling them to achieve long-term survival [5, 6]. According to the current application of multiple randomized controlled trials, PD-L1^+^ patients can benefit more from immunotherapy, and the degree of benefit is positively correlated with PD-L1 expression level [7]. The 4-year overall survival rate of patients with PD-L1 ≥ 1% was higher than that of PD-L1^−^ patients (>5%) [8], corresponded with the response rate to immunotherapy, 64% of PD-L1^+^ patients had a response to PD-1 monoclonal antibody, compared with 31% of PD-L1^−^ patients [9]. Moreover, response rates to ICIs have been reported to be only approximately 20% among patients with NSCLC in a population not screened for PD-L1 staining [10]. Therefore, there is an urgent need to explore therapeutic strategies for patients with PD-L1 negative NSCLC.

Sialic acid-binding immunoglobulin-like lectin 15 (Siglec-15) can promote osteoclast differentiation and increase bone resorption during bone remodeling [11, 12]. In 2019, Siglec-15 was identified as a novel immune checkpoint molecule that is less co-expressed with PD-L1 [13, 14]. Protein analysis showed that the extracellular domain encoded by the Siglec-15 gene shared more than 30% sequence homology with the B7 gene family encoding PD-L1, indicating that its potential immunoregulatory function was similar to that of B7 family members. Chen et al. found that Siglec-15 inhibited the immune response of antigen-specific T cells by affecting the expansion of T cells [13]. In vivo, Siglec-15 gene ablation inhibited the tumor growth rate, and the number of infiltrating CD8^+^T cells and NK cells and the production of IFN-γ and other cytokines were also significantly increased in the meantime.

In addition to acting as an immune suppressor, Siglec-15 can also act as a potential target for normalization cancer immunotherapy. Siglec-15 can be blocked by antibodies (NC-318), similar to immune checkpoint inhibitors for PD-1/PD-L1 and CTLA-4 [15]. Targeting Siglec-15, a new blocking antibody PYX-106 is currently in a phase I clinical study involving patients with advanced solid tumors (NCT04699123). PYX-106 is a fully human-derived anti-Siglec-15 monoclonal antibody with 10-fold higher affinity, and longer half-life (7 days vs. 1 day) compared with NC-318, and PYX-106 has a linear response to Siglec-15 inhibition in PBMC.

Our group has previously analyzed the expression profile, prognostic value, immune infiltration pattern, and potential biological function of Siglec-15 [16]. The results showed that Siglec-15 was upregulated in various tumors, including colon, thyroid, kidney, liver, and lung cancer. And expression of Siglec-15 was positively correlated with stromal CD8^+^ T cells infiltration [17]. Siglec-15 is specifically expressed in tumor cells and tumor-associated macrophages (TAMs). PD-L1 expression is present in approximately one-third of metastatic cancers, and the level of PD-L1 expression varies widely according to the type of primary tumor, the organ with distant metastasis, and other influencing factors, such as the treatment of chemotherapy or targeted therapy [18, 19]. At present, whether immunosuppression and poor prognosis induced by Siglec-15 are independent of PD-L1 remains unclear. Based on paraffin-embedded tissue microarray and multiplexed immunofluorescence staining to evaluate the expression of Siglec-15/PD-L1 and immune cell infiltration in the microenvironment, we reported that the expression of Siglec-15 and PD-L1 are independent. Subsequent stratified immune microenvironment analysis showed that PD-L1 negative patients were inhibited by Siglec-15^+^ macrophages, preventing CD8^+^T cell entry into the tumor, potentially leading to a poor prognosis.

## Materials and methods

### Patients and cohorts

A total of 135 patients underwent R0 resection and lymph node dissection from February 2013 to December 2014 with NSCLC were retrospectively collected in primary cohort (Table 1). None of the patients received antitumor treatments before surgery, and patients received postoperative radiotherapy or chemotherapy at the Tianjin Medical University Cancer Institute & Hospital. Tissue microarray were constructed as described previously [20]. In addition, we collected a validation cohort, which was purchased from Shanghai Outdo Biotech (Shanghai, China), including 50 LUAD samples without lymph node metastasis. Clinical information is based on the 8th edition of TNM system. Patients were followed up postoperatively every 3 months for the first 2 years, every 6 months for the next 3 years, and annually thereafter.

**Table 1:** Association of Siglec-15 and PD-L1 expression with clinical characteristics in the primary cohort.

		Siglec-15			PD-L1		
Characteristic	Total (*n* = 135), %	Negative	Positive	*p* value	Negative	Positive	*p* value
Age(years)							
<60	66(48.89)	55	11	0.09	36	30	0.91
≥60	69(51.11)	49	20		37	32	
Gender							
Male	74(54.81)	55	19	0.41	30	44	0.001***
Female	61(45.19)	49	12		43	18	
Clinical stage							
I	80(59.26)	62	18	0.99	45	35	0.27
II	21(15.55)	16	5		8	13	
IIIa	34(25.19)	26	8		20	14	
T classification							
T1	68(50.37)	55	13	0.27	40	28	0.52
T2	51(37.78)	35	16		26	25	
T3	13(9.63)	11	2		5	8	
T4	3(2.22)	3	0		2	1	
N classification							
N0	96(71.11)	73	23	0.15	52	44	0.80
N1	11(8.15)	11	0		5	6	
N2	28(20.74)	20	8		16	12	
Type							
LUAD	102(75.56)	77	25	0.45	64	38	<0.001***
LUSC	33(24.44)	27	6		9	24	

***p < 0.001.

Bold values indicates *p* < 0.05.

### Immunohistochemistry (IHC)

IHC was performed on NSCLC whole tissue sections, which were assigned by experienced pathologists. The following primary antibodies were used: CD8 (MA5-14548, Invitrogen), CD4 (ab133616, abcam), FoxP3 (14-4776-82, Invitrogen), Siglec-15 (BF8008, Affinity Bioscience), PD-L1 (MA5-27896, Invitrogen) and CD68 (14-0688-82, Invitrogen).

### Multiplexed immunofluorescence

Multiplexed immunofluorescence staining was performed based on the manufacturer’s protocol (PerkinElmer, Opal® Kit) to visualize cell markers. Slides were scanned and imaged using the Vectra Polaris Automated Quantitative Pathology Imaging System (PerkinElmer, Massachusetts, USA) at 200× magnification with the same exposure times. In brief, staining included multiple cycles of antigen retrieval (15 min boiling in antigen retrieval buffer, pH 6 or pH 9 depending on primary antibodies) followed by cooling, blocking, and consecutive staining with primary antibodies, HRP-polymer, and Opal fluorophores; cycles were repeated until all markers were stained. Finally, nuclei were stained with DAPI.

### Panel design

First Panel 1. Siglec-15 (BF8008, Affinity Bioscience, 1:500)—OPAL 520; 2. PD-L1 (MA5-27896, Invitrogen, 1:250)—OPAL 570; 3. CD68 (14-0688-82, Invitrogen, 1:250)—OPAL 620; 4. Pan-cytokeratin (CK) (ab27988, abcam, 1:200)—OPAL 650; 5. DAPI.

Second Panel 1. CD4 (ab133616, abcam, 1:500)—OPAL 480; 2. CD8 (MA5-14548, Invitrogen, 1:350)—OPAL 620; 3. FoxP3 (14-4776-82, Invitrogen, 1:100)—OPAL 650; 4. Siglec-15 (BF8008, Affinity Bioscience, 1:500)—OPAL 520; 5. PD-L1 (MA5-27896, Invitrogen, 1:250)—OPAL 570; 6. CD68 (14-0688-82, Invitrogen, 1:250)—OPAL 780; 7. Pan-CK (ab27988, abcam, 1:200)—OPAL 690; 8. DAPI.

### Multispectral imaging and immunophenotypes analysis

Multispectral images unmixing was performed using PerkinElmer inForm Image Analysis software (version 2.6.0). We divided the total tissue into tumor area and stroma area based on Pan-CK staining. Cells were typed according to our markers of interest as follows: tumor cells (Pan-CK^+^), non-tumor cells (Pan-CK^−^), cytotoxic T cells (CD8^+^), T regulatory cells (Tregs) (CD4^+^FoxP3^+^), tumor-associated macrophages (TAMs) (CD68^+^).

Following whole slide scans using inForm, at least three stamps (regions of interest; resolution: 2 pixels/ μm^2^; pixel size: 0.5 × 0.5 μm^2^) were set in non-necrotic areas. Enumerations at total, stroma and tumor area were summarized for all stamps per sample [21]. Phenotypes were determined according to median CD8^+^T cells density as follows: inflamed, ≥300 cells/mm^2^ at total area and ratio between stroma and tumor <2; excluded, ≥300 cells/mm^2^ at total area and ratio between the stroma and tumor ≥2; ignored, <300 cells/mm^2^ at total area. All scans fulfilled either of these 3 immunophenotypes.

### Cell culture and animals

The murine cell line TC-1, LLC and RAW264.7 were cultured at 37 °C in a humidified atmosphere of 95% air and 5% CO_2_ with DMEM, or RPMI-1640 basic medium supplemented with 10% FBS as medium. All cell lines were obtained from ATCC by STR profiling and tested negative for mycoplasma contamination. For mouse Siglec-15 stable expression cell lines, mouse Siglec-15 cDNA (NM_ 001101038.2) was cloned into pLV-Siglec-15. pLV vector was used as control. Lentiviral infections were performed according to standard procedures.

Female C57BL/6 mice of 4 weeks were used in animal experiments, and mice were maintained in the specific pathogen-free conditions. Each group was randomly divided into 5 mice. A total of 1 × 10^6^ LLC cells and 3 × 10^5^ vector or pLV-Siglec-15 transfected RAW cells were subcutaneously injected in a blinded manner. After 4 weeks, mice were killed and the tumors were collected to make tissue sections.

### Quantitative real-time PCR

Total RNA was extracted with TRIZOL Reagent (Invitrogen, Carlsbad, CA, USA). Complementary DNA was prepared by reverse-transcription as standard protocol described, followed by quantitative real-time PCR.

### Gene set enrichment analysis

We downloaded the FPKM data of LUAD from TCGA portal (https://portal.gdc.cancer.gov/). We performed GSEA to discover potential pathways by Kyoto Encyclopedia of Genes and Genomes (KEGG) and Hallmark terms affected by Siglec-15 using Sangerbox (http://www.sangerbox.com/tool).

### Statistical analysis

GraphPad Prism version 9.0 was used for graph drawing and statistical analysis. Kaplan–Meier curves were used and estimated by the log-rank test in Sangerbox (http://www.sangerbox.com/tool). Univariate and multivariate regression analyses was performed by Cox regression analysis. Variables with *p* ≤ 0.10 in the univariate analysis were subjected to multivariate analysis. Wilcoxon test was used to assess differences in immune cell densities; Pearson-correlation was used to assess linear relationships in normal distribution; and Chi-Square test or Fishers’ exact test (in case of small sample sizes) were used to assess relationships among factorial variables. The following significance levels were used: *p < 0.05; **p < 0.01; ***p < 0.001; NS, *p* > 0.5.

## Results

### Definition of Siglec-15 and PD-L1 positivity in NSCLC tissues

Analysis of multiplex staining images from the NSCLC cohort showed that Siglec-15 and PD-L1 could be detected in both tumor cells (TC) and non-tumor cells (NTC; Fig. S1A). Quantification of immunofluorescence signals showed that the staining percentage of Siglec-15 on macrophages (Mφ-Siglec-15) was significantly higher than that of Siglec-15 on tumor cells (TC-Siglec-15) (8.73%; 95%CI, 6.65–10.81%; *p* < 0.001) (Fig. S1B). There was no significant difference in the staining percentage of PD-L1 on non-tumor cells (NTC-PD-L1) versus PD-L1 on tumor cells (TC-PD-L1) (Fig. S1C). The expression of TC-Siglec-15 was positively correlated with Mφ-Siglec-15 (*r* = 0.68; *p* < 0.001; Fig. S1D), but weakly correlated with TC-PD-L1 (*r* = 0.25; *p* = 0.003; Fig. S1E). The expression of NTC-PD-L1 was positively correlated with TC-PD-L1 (*r* = 0.85; *p* < 0.001; Fig. S1F), but weakly correlated with Mφ-Siglec-15 (*r* = 0.20; *p* = 0.02; Fig. S1G).

Based on the optimal cutoff values of expression of Siglec-15 and PD-L1, 12 patients (8.89%) were defined as TC-Siglec-15^+^, 31 patients (22.96%) were Mφ-Siglec-15^+^, and 57 patients (42.22%) were TC-PD-L1^+^, 54 patients (40.00%) were NTC-PD-L1^+^ in the cohort (Fig. S2). In NSCLC tissues, the co-expression of Siglec-15 and PD-L1 on TC was 9 (6.67%), and the co-expression of Mφ-Siglec-15 and NTC-PD-L1 was 15 (11.11%) (Fig. S1E, G).

### Siglec-15 and PD-L1 expression were independent

**Fig. 1:**
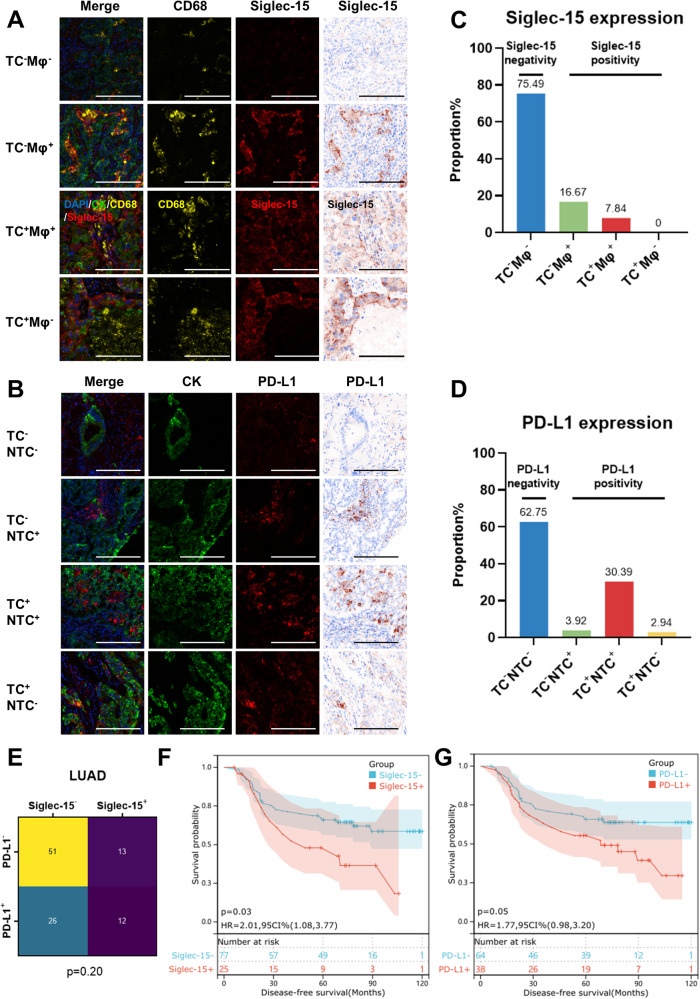
Different expression patterns of Siglec-15 and PD-L1 in LUAD. **A** Representative images of NSCLC tissue sections with Siglec-15 expression Pattern 1 (TC^−^Mφ^−^), Pattern 2 (TC^−^Mφ^+^), Pattern 3 (TC^+^Mφ^+^), and Pattern 4 (TC^+^Mφ^−^). Scale bar, 50 μm. **B** Representative images of NSCLC tissue sections with PD-L1 expression Pattern 1 (TC^−^NTC^−^), Pattern 2 (TC^−^NTC^+^), Pattern 3 (TC^+^NTC^+^), and Pattern 4 (TC^+^NTC^-^). Scale bar, 50 μm. **C**, **D** LUAD samples were divided into four groups according to their Siglec-15 or PD-L1 expression patterns. **E** Chi-Square test showed the relationship between Siglec-15 and PD-L1. **F**, **G** Comparison of DFS between patients with Siglec-15 or PD-L1 positivity and negativity.

Using multispectral images of Siglec-15, Pan-CK, and CD68, we divided NSCLC samples into four groups according to their Siglec-15 positivity patterns (Fig. 1A): TC-Siglec-15^+^ and Mφ-Siglec-15^+^, TC-Siglec-15^+^ and Mφ-Siglec-15^−^, TC-Siglec-15^−^ and Mφ-Siglec-15^+^, TC-Siglec-15^−^ and Mφ-Siglec-15^−^, and we identified patients with positive Siglec-15 expression on macrophages or TC as Siglec-15^+^. As shown in Fig. S3A, 104 patients (77.04%) were Siglec-15^−^, and 31 patients (22.96%) were classified as Siglec-15^+^, including 12 (8.89%) with TC and macrophages positivity, and 19 (14.07%) with macrophages positivity only. Patients were also divided into four groups according to PD-L1 expression (Fig. 1B), and we defined patients with positive PD-L1 expression on NTC or TC as PD-L1^+^, 73 patients (54.07%) were PD-L1^−^, and 62 patients (45.93%) were classified as PD-L1^+^, including 49 (36.30%) with NTC and TC positivity, 8 (5.93%) with TC positivity only, and 5 (3.70%) with NTC positivity only (Fig. S3B). Furthermore, Siglec-15 positivity was found to be independent of PD-L1 positivity (*p* = 0.26; Fig. S3C).

We further analyzed Siglec-15 and PD-L1 expression patterns in different pathological subtypes. There were 102 lung adenocarcinoma (LUAD) patients in the cohort, 77 patients (75.49%) were negative for Siglec-15 expression, and 25 patients (24.51%) were classified as Siglec-15^+^, including 8 (7.84%) with TC and macrophages positivity, and 17 (16.67%) with macrophages positivity only (Fig. 1C). 64 patients (62.75%) were negative for PD-L1 expression, and 38 patients (37.25%) were classified as PD-L1^+^, including 31 (30.39%) with NTC and TC positivity, 3 (2.94%) with TC positivity only, and 4 (3.92%) with NTC positivity only (Fig. 1D). Meanwhile, we found similar Siglec-15 positive rate and higher PD-L1 positive rate in LUSC patients compared with LUAD, and Siglec-15 positivity and PD-L1 positivity in different pathological subtypes were independent (*p* > 0.05, Fig. 1E, S4A–C).

### Siglec-15 and PD-L1 predict DFS in non-metastasis LUAD

The median age of this cohort was 60 years (range, 37–79 years), and the median follow-up for survivors was 74.3 months (range, 6.1–120.2 months). The 5-year OS and DFS rates of the entire cohort were 81.48% and 64.44%, respectively. We found that Siglec-15/PD-L1 positivity did not alter disease-free survival (DFS) in the entire NSCLC cohort (Fig. S3D, E), a result that may have been influenced by LUSC patients (Fig. S4D, E). We further analyzed the LUAD patients, Siglec-15^+^ patients had worse DFS (hazard ratio [HR], 2.01; 95%CI, 1.08–3.77; *p* = 0.03) (Fig. 1F). PD-L1 positivity was significantly associated with worse DFS (HR, 1.77; 95% CI, 0.98–3.20; *p* = 0.05) (Fig. 1G). Multivariate analysis further showed (Table 2) that Siglec-15 positivity was independently associated with DFS (HR, 1.87; 95%CI, 1.00–3.51; *p* = 0.05), and N classification was also an independent prognostic factor for DFS (HR, 3.92; 95%CI, 2.15–7.17; *p* < 0.001). Considering that Siglec-15 and PD-L1 were not prognostic factors for LUSC patients, we mainly analyzed LUAD patients in the subsequent.

**Table 2:** Univariate and multivariate analyses for disease-free survival and overall survival in LUAD.

	Disease-free survival				Overall survival			
	Univariate		Multivariate		Univariate		Multivariate	
Characteristic	Patients no./total no. (%)	*p* value	Hazard ratio (95%CI)	*p* value	Patients no./total no. (%)	*p* value	Hazard ratio (95%CI)	*p* value
Age(years)								
<60	20/54(37.04)	0.19	NA	NA	8/54(14.81)	0.15	NA	NA
≥60	24/48(50.00)				13/48(27.08)			
Gender								
Male	20/46(43.48)	0.86	NA	NA	10/46(21.74)	0.68	NA	NA
Female	24/56(42.86)				11/56(19.64)			
Clinical stage								
I	19/64(29.69)	<0.001***	NA	NA	8/64(12.5)	<0.001***	NA	NA
II	6/12(50.00)				2/12(16.67)			
IIIa	19/26(73.08)				11/26(42.31)			
T classification								
T1	22/61(36.07)	0.04*	NA	NA	9/61(14.75)	0.03*	NA	NA
T2+T3+T4	22/41(53.66)				12/41(29.27)			
N classification								
N0	22/72(30.56)	<0.001***	**3.92**	<0.001***	10/72(13.89)	<0.001***	NA	NA
N1+N2	22/30(73.33)		**(2.15–7.17)**		11/30(36.67)			
Siglec-15								
Negative	29/77(37.66)	0.03*	**1.87**	**0.05**	14/77(18.18)	0.14	NA	NA
Positive	15/25(60.00)		**(1.00–3.51)**		7/25(28.0)			
PD-L1								
Negative	22/64(34.38)	0.05*	NA	NA	10/64(15.63)	0.15	NA	NA
Positive	22/38(57.89)				11/38(28.95)			

*p < 0.05, and ***p < 0.001

Bold values indicates *p* < 0.05.

**Fig. 2:**
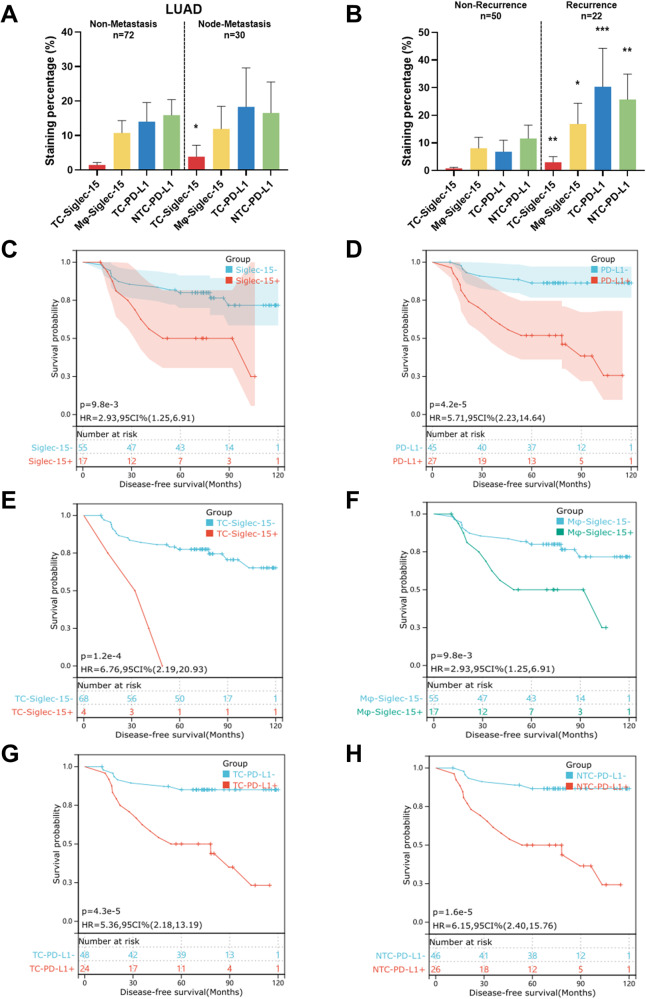
Siglec-15 and PD-L1 predict DFS in non-metastasis LUAD. **A** Comparison of the staining percentage of Siglec-15 and PD-L1 in patients with and without lymph node metastasis. The bar indicates mean with 95% CI. **B** Comparison of the staining percentage of Siglec-15 and PD-L1 in patients without lymph node metastasis developed recurrence and non-recurrence. The bar indicates mean with 95% CI. **C**, **D** Comparison of DFS between non-metastasis LUAD patients with Siglec-15/PD-L1 positivity and negativity. **E**, **F** Comparison of DFS between non-metastasis LUAD patients with TC-Siglec-15 or Mφ-Siglec-15 positivity and negativity. **G**, **H** Comparison of DFS between non-metastasis LUAD patients with TC-PD-L1 or NTC-PD-L1 positivity or negativity. *p < 0.05, **p < 0.01 and ***p < 0.001.

Table 1 showed that *N* classification and Siglec-15 were independent prognostic factors. We further explored the effect of Siglec-15 on the prognosis of patients with different *N* classifications. The staining percentage of TC-Siglec-15 in 30 patients (29.41%) with lymph node metastasis were significantly lower than that in 72 patients (70.59%) without lymph node metastasis (median [range], 1.49 [0–19.09] vs 3.88 [0–42.72]; *p* = 0.05; Fig. 2A). We further found that the staining percentage of TC-Siglec-15 (median [range], 3.01 [0.04–19.09] vs 0.82 [0–5.67]; *p* = 0.003) and Mφ-Siglec-15 (median [range], 16.89 [0.51–48.64] vs 8.10 [0–61.31]; *p* = 0.02) were significantly higher in non-metastasis LUAD patients who developed recurrence than in those who hadn’t developed recurrence (Fig. 2B). The results suggested that Siglec-15 expression could predict the prognosis of patients without metastasis.

Kaplan–Meier analysis showed that Siglec-15^+^ patients without metastasis had worse DFS (HR, 2.93; 95% CI, 1.25–6.91; *p* = 0.01), and PD-L1 positivity had a significant effect on DFS (HR, 5.71; 95%CI, 2.23–14.64; *p* < 0.001) (Fig. 2C, D). Univariate survival analysis of Siglec-15/PD-L1 in each tumor part showed that TC-Siglec-15^+^ patients had worse DFS (HR, 6.76; 95%CI, 2.19–20.93; *p* < 0.001), and Mφ-Siglec-15^+^ patients had worse DFS (HR, 2.93; 95% CI, 1.25–6.91; *p* = 0.01), TC-PD-L1 (HR, 5.36; 95%CI, 2.18–13.19; *p* < 0.001) and NTC-PD-L1 (HR, 6.15; 95% CI, 2.40–15.76; *p* < 0.001) was also unfavorable for DFS (Fig. 2E–H).

### Siglec-15 could increase the risk of recurrence in PD-L1^−^ patients

**Fig. 3:**
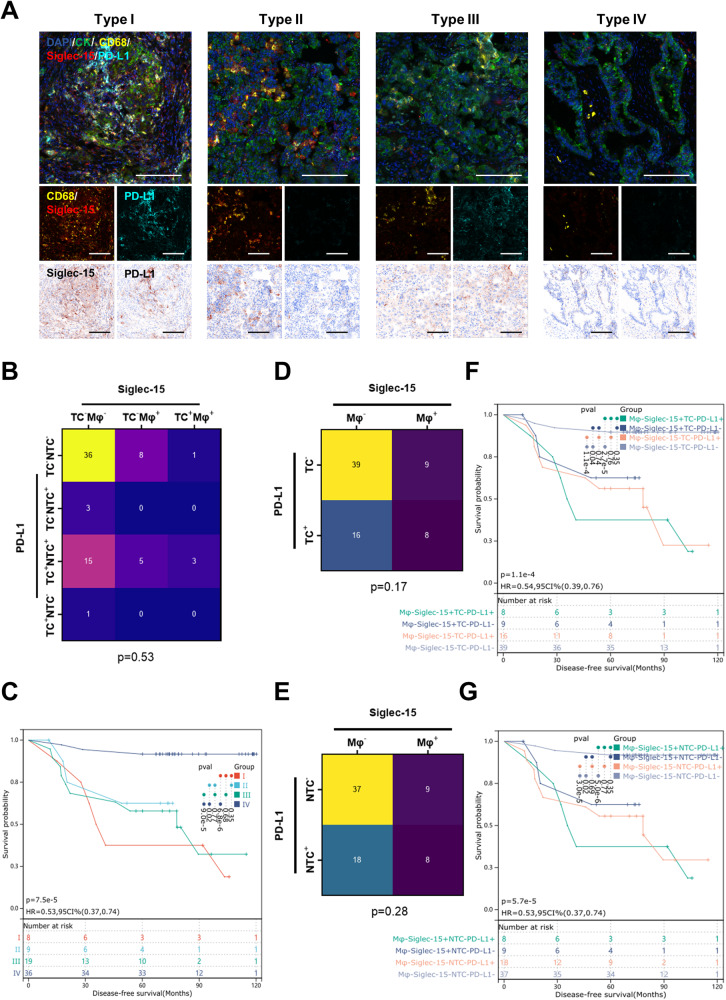
Association of checkpoint types with DFS. **A** Representative images of LUAD tissue sections with checkpoint type I (Siglec-15 positivity with PD-L1 positivity), type II (Siglec-15 positivity with PD-L1 negativity), type III (Siglec-15 negativity with PD-L1 positivity), and type IV (Siglec-15 negativity with PD-L1 negativity). Scale bar, 50 μm. **B** Chi-Square test showed the relationship between Siglec-15 and PD-L1 patterns. **C** Comparison of DFS by different types in non-metastasis LUAD patients. **D**, **E** Chi-Square test showed the relationship between Mφ-Siglec-15 and TC-PD-L1/NTC-PD-L1. **F**, **G** Comparison of DFS among Mφ-Siglec-15 and TC-PD-L1/NTC-PD-L1 patterns in non-metastasis LUAD patients.

Considering the expression patterns of Siglec-15 and PD-L1, we classified the 72 LUAD patients without metastasis into four checkpoint types (Fig. 3A): type I (Siglec-15^+^PD-L1^+^; 8 patients [11.11%]), type II (Siglec-15^+^PD-L1^−^; 9 patients [12.50%]), type III (Siglec-15^−^PD-L1^+^; 19 patients [26.39%]) and type IV (Siglec-15^−^PD-L1^−^; 36 patients [50.00%]), with corresponding recurrence rates of 6 patients (75.00%), 3 patients (33.33%), 10 patients (52.63%), and 3 patients (8.33%), respectively. Siglec-15 and PD-L1 expression were independently distributed in the four types (*p* = 0.53; Fig. 3B). Survival analysis showed that type I patients had the worst prognosis and type IV patients had the best DFS (*p* < 0.001; Fig. 3C). Moreover, compared with type IV, type II and III patients had significantly worse DFS (*p* = 0.02; *p* < 0.001). The 5-year DFS rates of type I, II, III, and IV were 37.50%, 66.67%, 57.89%, and 91.67%, respectively.

Figure 3B showed that Siglec-15 is mainly expressed by macrophages and that Mφ-Siglec-15 expression is independent of PD-L1 expression (*p* < 0.05; Fig. 3D, E), and we revealed that Siglec-15 expressed on TAMs showed more important prognostic value than that expressed on tumor cells in previous study [17], thus we further analyzed the effect of Mφ-Siglec-15 and PD-L1 expression on the prognosis of patients. Consistent with the results of survival analysis in Fig. 3C, Mφ-Siglec-15^+^PD-L1^+^ patients had the worst prognosis, and Mφ-Siglec-15^−^PD-L1^−^ patients had the best prognosis (*p* < 0.001). Furthermore, in TC-PD-L1^−^ (*p* = 0.04; Fig. 3F) or NTC-PD-L1^−^ (*p* = 0.02; Fig. 3G) patients, Mφ-Siglec-15 positivity was associated with a significantly increased risk of DFS. The results showed that the expression of Mφ-Siglec-15 was of great significance for PD-L1^−^ patients.

### Siglec-15^+^ macrophages were negatively associated with inflamed immunophenotype in PD-L1^−^ patients

**Fig. 4:**
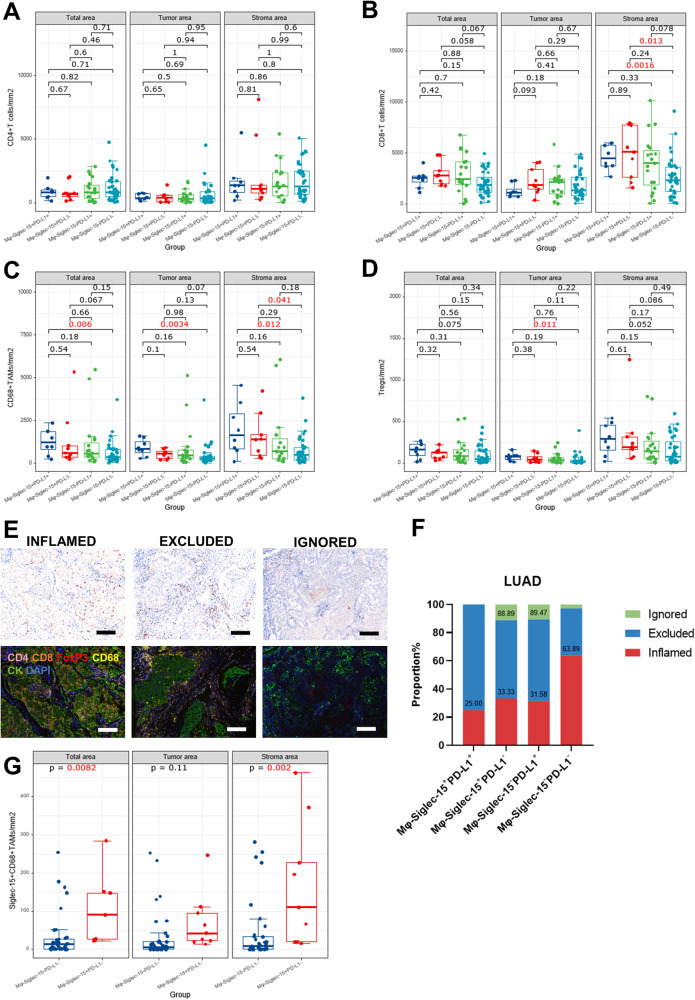
Siglec-15^+^ macrophages were negatively associated with inflamed immunophenotype in PD-L1^−^ patients. Among Mφ-Siglec-15 and PD-L1 patterns in non-metastasis LUAD patients, box plot showed the density of **A** CD4^+^T cells, **B** CD8^+^T cells, **C** CD68^+^TAMs and **D** CD4^+^FoxP3^+^Tregs. The bar indicates mean with 95% CI. **E** Representative slide images of CD8^+^T cell immunophenotypes with the percentage of patients per phenotype and representative multiplex IF images of immune effector cells at tumor and stroma area of each phenotype. Scale bar, 50 μm. **F** The proportions of immunophenotypes among expression patterns of Mφ-Siglec-15 and PD-L1. **G** Comparison of density of Siglec-15^+^CD68^+^TAMs between Mφ-Siglec-15^−^PD-L1^−^ and Mφ-Siglec-15^+^PD-L1^−^LUAD patients. The bar indicates mean with 95% CI.

We further investigated the infiltration of immune cells in the microenvironment of LUAD patients. The infiltration density of CD68^+^TAMs and CD4^+^FoxP3^+^Tregs in the tumor area of Mφ-Siglec-15^+^PD-L1^+^ patients were higher than that of Mφ-Siglec-15^−^PD-L1^−^ patients (*p* < 0.05). Mφ-Siglec-15^+^PD-L1^−^ patients had more CD8^+^T cells than Mφ-Siglec-15^−^PD-L1^−^ patients in the stroma (*p* = 0.013; Fig. 4A–D), the total CD8^+^T cells infiltration density did not differ among the four types (Fig. S5). The role of Mφ-Siglec-15 on the microenvironment needs to be further explored.

Considering the different distribution characteristics of CD8^+^T cells in the tumor area and stroma area, three immunophenotypes were defined on the basis of the distribution of CD8^+^T cells: inflamed (34 patients [47.22%]; CD8^+^T cells were evenly distributed in the stroma and tumor area); excluded (34 patients [47.22%]; CD8^+^T cells were mainly located in the stroma, not in the tumor area) and ignored (4 patients [5.56%]; fewer CD8^+^T cells were found in both stroma and tumor area) (Fig. 4E), and corresponding recurrence rates were 7 patients (20.58%), 13 patients (38.24%) and 2 patients (50%), respectively. The density and distribution of CD8^+^T cells in the three immunophenotypes were significantly different (*p* < 0.01; Fig. S6A–D).

We found that the proportion of inflamed phenotype of Mφ-Siglec-15^−^PD-L1^−^ patients was significantly higher than that of other patients, and there was no significant difference among the other patients (63.89% vs 25% vs 33.33% vs 31.58%; Fig. 4F), and the proportion of ignored phenotype of Mφ-Siglec-15^−^PD-L1^−^ patients was lower than Mφ-Siglec-15^+^PD-L1^−^ patients. Further analysis of the distribution of Siglec-15^+^CD68^+^TAMs in PD-L1^−^ patients showed that stromal Siglec-15^+^CD68^+^TAMs of Mφ-Siglec-15^+^PD-L1^−^ patients were significantly more than those of Mφ-Siglec-15^−^PD-L1^−^ patients (*p* = 0.002; Fig. 4G), suggesting that stromal Siglec-15^+^CD68^+^TAMs may inhibit the infiltration of CD8^+^T cells into the tumor.

### Siglec-15^+^ macrophages inhibit CD8^+^T cells infiltration

**Fig. 5:**
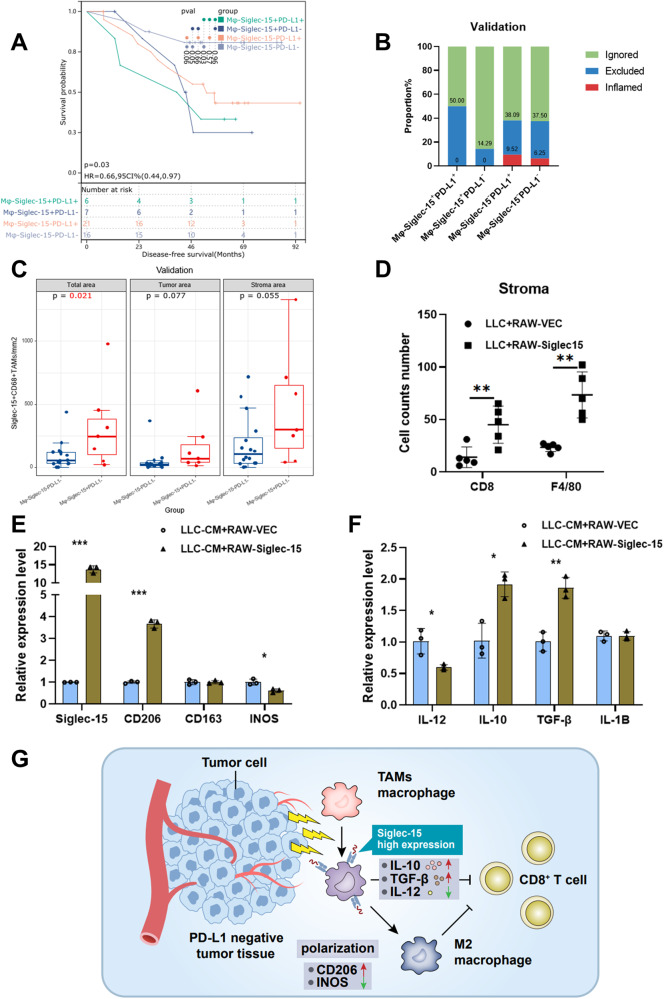
Siglec-15^+^ macrophages inhibit CD8^+^T cells infiltration. **A** Comparison of disease-free survival among Mφ-Siglec-15 and PD-L1 patterns in the validation cohort. **B** The proportions of immunophenotypes among expression patterns of Mφ-Siglec-15 and PD-L1. **C** Comparison of density of Siglec-15^+^ TAMs between Mφ-Siglec-15^−^PD-L1^−^ and Mφ-Siglec-15^+^PD-L1^−^ patients in the validation cohort. The bar indicates mean with 95% CI. **D** Comparison of number of stromal immune cells between two groups in vivo. There were 5 mice in each group, a total of 1 × 10^6^ LLC cells and 3 × 10^5^ vector or pLV-Siglec-15 transfected RAW cells were subcutaneously injected in each mouse. The bar indicates mean with SD. **E**, **F** The mRNA level of macrophage markers and cytokines were measured by qPCR. The bar indicates mean with SD. The supernatants of LLC cells were collected to treat RAW-Siglec-15 cells for 48 h. This experiment was performed in triplicate and repeated three times with the same results. **G** Schematic diagram. Siglec-15-highly expressed TAMs infiltrate into PD-L1 negative tumor tissues and secrete some cytokines that inhibit CD8^+^ T cells, such as increased IL-10 and decreased IL-12 et al. In addition, these Siglec-15-highly expressed TAMs polarize toward the M2 type, further inhibiting CD8^+^ T cells infiltration. *p < 0.05, **p < 0.01 and ***p < 0.001.

Table S1 summarized the characteristics of the 50 non-metastasis LUAD patients in the validation cohort. During a median follow-up of 47 months (range, 7.3–94 months) in this cohort, 22 patients (44%) developed local or distant recurrences. According to the cutoff values defined in the main cohort, 13 patients (26.0%) were Mφ-Siglec-15^+^ and 27 patients (54.0%) were PD-L1^+^, independently of each other (*p* = 0.51; Fig. S7A). Kaplan–Meier curves confirmed the survival differences among the four types, Mφ-Siglec-15^+^PD-L1^+^ and Mφ-Siglec-15^+^PD-L1^−^ patients had significantly lower DFS rates than Mφ-Siglec-15^−^PD-L1^−^ patients (*p* = 0.03; *p* = 0.05; Fig. 5A).

In microenvironment, the proportion of ignored phenotype of Mφ-Siglec-15^−^PD-L1^−^ patients was significantly lower than that of Mφ-Siglec-15^+^PD-L1^−^ patients (62.50% vs 85.71%; Fig. 5B, S7B), and there was a trend toward more stromal Siglec-15^+^CD68^+^TAMs in Mφ-Siglec-15^+^ patients (Fig. 5C). Furthermore, we mixed low-PD-L1 expressed murine lung cancer cell line LLC and Siglec-15 overexpressed RAW cells to inject mice (Fig. S7C), and the infiltration of immune cells was detected by IHC (Fig. S7D). We found that the number of CD8^+^T cells in the stroma area of the overexpression Siglec-15 group was higher than that of the control group (*p* = 0.009; Fig. 5D, S7E). At the same time, we observed more F4/80^+^TAMs in the stroma area of the overexpression Siglec-15 group (*p* = 0.001). We observed the immunosuppressive effect of Siglec-15^+^ macrophages in vivo, consistent with what was found in patients.

To explore the underlying mechanisms, we examined macrophage phenotype and cytokines production in a co-culture system. Upon LLC-condition media (CM) stimulation, Siglec-15^+^ macrophages were polarized toward M2 phenotype with increased CD206 and decreased INOS (Fig. 5E). Meanwhile, the expression of IL-10 and TGF-β were increased and IL-12 was decreased in Siglec-15^+^ macrophages (Fig. 5F). These results suggest that the polarization trend and increased expression of immunosuppressive cytokines of Siglec-15^+^ macrophages may inhibit CD8^+^T cells infiltration. In addition, analysis in TCGA database found that NOD-LIKE and TOLL-LIKE receptor signaling pathways were upregulated in Siglec-15-H + PD-L1-L patients (Fig. S8A). Hallmark enrichment analysis showed that TNF-α, IL-2 and IL-6 signals were upregulated in Siglec-15-H + PD-L1-L patients compared with Siglec-15-L + PD-L1-L patients (Fig. S8B). These immune-related signaling pathways are related to the production of cytokines or activation of cytokine downstream signals in the microenvironment. We further found that MCSF, IL-1β and TNF-α were positively correlated with the expression of Siglec-15 (Fig. S8C). These results suggest that these cytokines in the microenvironment may be involved in the regulation of Siglec-15 expression. Schematic diagram showed the key discovery, we initially outlined the mechanism by which Siglec-15^+^ macrophages inhibit the microenvironment. (Fig. 5G).

## Discussion

Specific monoclonal antibodies that block the PD-1/PD-L1 pathway are widely used in current ICI therapy, and these kinds of immunotherapy successfully normalize immune system in patients who respond to ICIs [22, 23]. However, the therapy targeting the PD-1/PD-L1 pathway has limitations, and the response rate of PD-L1 positive patients is much higher than that of PD-L1 negative patients [24, 25]. Siglec-15 is an exciting novel immune checkpoint molecule, and the co-expression rate of Siglec-15 and PD-L1 is only 3% [13]. This implies that Siglec-15 and PD-L1 may suppress the immune microenvironment through independent mechanisms. Drugs targeting Siglec-15 are expected to provide a new treatment option for patients who fail to respond to PD-1/PD-L1 antibody treatment [26]. A pioneer targeting Siglec-15 was NextCure’s NC-318 (Siglec-15 monoclonal antibody), which blocks Siglec-15-induced immunosuppression by targeting M2 macrophages and Siglec-15 positive tumor cells [14, 27]. Compared with NC-318, PYX-106 is a fully human derived antibody with a 10-fold higher affinity and a substantially prolonged half-life. Pyxis Oncology has received clinical approval in the United States and Europe and is in phase I clinical trials targeting a variety of solid tumors.

Another approach uses agents that target the immunoregulatory interaction between Siglecs and their sialic acid ligands to reprogram immune cells for an immunologic attack [28]. The sialic acid-targeting approach furthest along in clinical development for involves degradation of overexpressed sialic acid moieties on tumor cells with a technology termed “EAGLE” (Enzyme-Antibody Glycan-Ligand Editing). The technology was originally developed by combining an anti-HER2 to a sialidase conjugate that selectively removed diverse sialoglycans from breast cancer cells, leading to enhanced immune cell infiltration and activation, as well as prolonged survival, in mouse models [29].

The expression of Siglec-15 is one of the core issues of ICI therapy targeting Siglec-15. Our research group had conducted a pan-cancer study of Siglec-15 in 2020 which showed the expression of Siglec-15 in different cancer types and its influence on prognosis from TCGA, GEO and GTEx database. That study showed that the upregulated expression of Siglec-15 in LUAD is positively associated with poor OS [16]. The results of our present study showed that the co-expression rates of Siglec-15 and PD-L1 are 6.67% in NSCLC, which are similar to those reported by Wang et al. [13], in which they reported that Siglec-15 and PD-L1 were positive for both markers in only approximately 3% of specimens in NSCLC. However, the expression patterns and prognostic value of Siglec-15 and PD-L1 in other tumors are different from those in NSCLC. Recent study reported that the co-expression rates of Siglec-15 and PD-L1 on TC and macrophages are 30.0% and 22.3% in esophageal squamous cell carcinoma (ESCC) patients, respectively. And Siglec-15 represented good prognosis in the esophageal cancer cohort, contrary to that in NSCLC [30]. In addition, Fudaba et al. found that co-expression of Siglec-15 and PD-L1 was detected in approximately 20% of macrophages in lymphomas [31]. There may be some potential reasons for the difference. Firstly, treatment regimens differed among the study cohorts, which may have had some effect on immune checkpoint expression. Our study cohort was newly diagnosed lung cancer patients who underwent surgery, and the esophageal cancer cohort was patients who received neoadjuvant chemoradiotherapy.

The heterogeneous expression of Siglec-15 in different tumor types may induce different immune microenvironments. For example, Siglec-15 was positively correlated with CD8^+^T cells in adrenocortical carcinoma but negatively correlated with CD8^+^T cells in BRCA-based breast cancer [16]. Recently we found that Siglec-15 was positively with CD8^+^T cells infiltration in the stroma area of LUAD. Spatially, Siglec-15^+^TAMs infiltrated with more CD8^+^T cells and were closer to CD8^+^T cells than Siglec-15^+^tumor cells, which may play a major role in the interaction with CD8^+^T cells [17].

Wang et al. used T cell activity array (TCAA) to reveal the role of Siglec-15 in suppressing antitumor immunity, but the specific site of CD8^+^T cells binding to Siglec-15 was not identified. In 2023, nuclear magnetic resonance spectroscopy and molecular dynamics simulations demonstrate that binding of Siglec-15 to T cells depends on the presence of sialoglycans, and identify the leukocyte integrin CD11b as a Siglec-15 binding partner on human T cells [32]. These results finally yielded the structural basis of Siglec-15 inhibitory pathway, which provided guidance for the study of signal transduction in immune cells.

In this study, we speculated that Siglec-15^+^TAMs formed a net and intercepted CD8^+^T cells to kill tumor cells after TAMs combined with CD8^+^T cells in stroma area. Patients with type II LUAD (Siglec-15 positivity with PD-L1 negativity) have worse outcomes and are most likely to benefit from a single anti-Siglec-15. And the strategy of combining anti-PD-1 and anti-Siglec-15 blockade might be appropriate for patients with type I disease (Siglec-15 positivity with PD-L1 positivity). Patients with type IV disease (Siglec-15 negativity with PD-L1 negativity) require additional immune checkpoint molecular need to be detected to guide treatment. In summary, our results indicate that PD-L1-independent Siglec-15^+^TAMs suppress immune microenvironment in non-metastasis LUAD patients. Siglec-15 could be an ICI target for low-PD-L1 expressed patients who will not have response to anti-PD-L1.

### Supplementary information

Supplementary material

## Data Availability

The data that support the findings of this study are available from the corresponding author (yanglili@tjmuch.com) upon reasonable request.
