# Supplementary material for: Siglec-15 on macrophages suppress the immune microenvironment in patients with PD-L1 negative non-metastasis lung adenocarcinoma
Source: Cancer Gene Ther. 2023 Dec 11;31(3):427–38. doi: 10.1038/s41417-023-00713-z (PMC10940158; doi:10.1038/s41417-023-00713-z)
Supplement: Supplementary file 1 — Supplementary material [file 41417_2023_713_MOESM1_ESM.pdf]

Supplemental Content

Table S1. Association of Siglec-15 and PD-L1 expression with clinical characteristics in the validation cohort.

Table S2. Primer sequences.

Fig. S1. Siglec-15 and PD-L1 expression in NSCLC tissues.

Fig. S2. Staining percentage of Siglec-15 and PD-L1 in NSCLC tissues.

Fig. S3. Different expression patterns of Siglec-15 and PD-L1 in NSCLC.

Fig. S4. Different expression patterns of Siglec-15 and PD-L1 in LUSC.

Fig. S5. Representative images of LUAD tissue section.

Fig. S6. Density of immune cells among the immunophenotypes.

Fig. S7. Validation of the immunophenotypes.

Fig. S8. Pathway analysis of Siglec-15 in LUAD from TCGA.

Table S1. Association of Siglec-15 and PD-L1 expression with clinical characteristics in the validation cohort.

Characteristic	Total (n = 50), %	Siglec-15			PD-L1		
		Negative	Positive	*p* Value	Negative	Positive	*p* Value
Age(years)							
<60	15(30.00)	13	2	0.18	8	7	0.5
≥60	35(70.00)	24	11		15	20	
Gender							
Male	27(54.00)	20	7	0.99	11	16	0.42
Female	23(46.00)	17	6		12	11	
Clinical stage							
I	38(76.00)	29	9	0.25	19	19	0.25
II	9(18.00)	7	2		2	7	
IIIa	3(6.00)	1	2		2	1	
T classification							
T1	18(36.00)	13	5	0.36	11	7	0.16
T2	22(44.00)	17	5		9	13	
T3	7(14.00)	6	1		1	6	
T4	3(6.00)	1	2		2	1	

*$p < 0.05$, **$p < 0.01$ and ***$p < 0.001$.

Table S2. Primer sequences.

Species	Target	Direction	Sequences
mouse	Cd274	forward	5-AGCCTCAGCACAGCAACTTCAG-3
		reverse	5-CTTGTAGTCCGCACCACCGTAG-3
mouse	Siglec15	forward	5-TGCTGCTGCTTGGCATTCTGG-3
		reverse	5-CCTGAGCCTGAGACCGTGGAG-3
mouse	Cd163	forward	5-AATCACATCATGGCACAGGTCACC-3
		reverse	5-TCGTCGCTTCAGAGTCCACAGG-3
mouse	Inos	forward	5-ACTCAGCCAAGCCCTCACCTAC-3
		reverse	5-TCCAATCTCTGCCTATCCGTCTCG-3
mouse	Cd206	forward	5-GGCGAGCATCAAGAGTAAAGA-3
		reverse	5-CATAGGTCAGTCCCAACCAAA-3
mouse	Il10	forward	5-TTCTTTCAAACAAAGGACCAGC-3
		reverse	5-GCAACCCAAGTAACCCTTAAAG-3
mouse	Tgfb1	forward	5-CCAGATCCTGTCCAAACTAAGG-3
		reverse	5-CTCTTTAGCATAGTAGTCCGCT-3
mouse	Il12	forward	5-TGAGAAGTATTCAGTGTCCTGC-3
		reverse	5-CTGTGAGTTCTTCAAAGGCTTC-3
mouse	Il1b	forward	5-CACTACAGGCTCCGAGATGAACAAC-3
		reverse	5-TGTCGTTGCTTGGTTCTCCTTGTAC-3

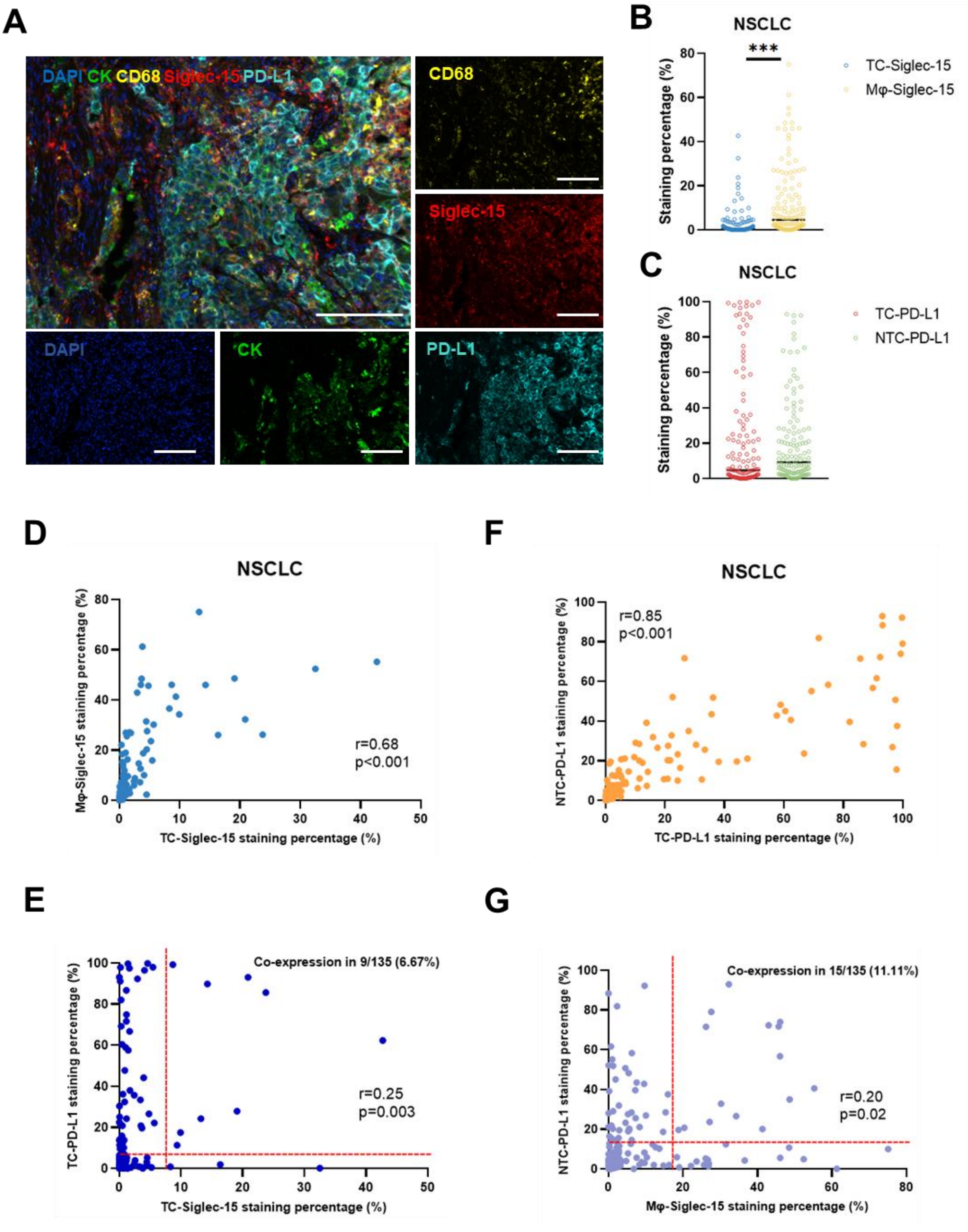

Fig. S1. Siglec-15 and PD-L1 expression in NSCLC tissues.

(A) Representative images of NSCLC tissue section by multiplex immunofluorescence staining with the indicated markers. Scale bar, 50μm. (B) Analysis of the percentages of Siglec-15 staining NSCLC tumor cells and macrophages. Tumor cells were identified as pan-cytokeratin positive, and macrophages were identified as CD68 positive. The bar indicates mean. (C) Analysis of the percentages of PD-L1 staining NSCLC tumor cells and non-tumor cells. Tumor cells were identified as pan-cytokeratin positive, and non-tumor cells were identified as pan-cytokeratin negative. The bar indicates mean. (D) Correlation between TC-

Siglec-15 and Mφ-Siglec-15 staining percentages on the same tumor sections. (E) The co-expression and correlation between TC-Siglec-15 staining percentage and TC-PD-L1 staining percentage on the same tumor sections. (F) Correlation between TC-PD-L1 and NTC-PD-L1 staining percentages on the same tumor sections. (G) The co-expression and correlation between Mφ-Siglec-15 staining percentage and NTC-PD-L1 staining percentage on the same tumor sections. *$p < 0.05$, **$p < 0.01$ and ***$p < 0.001$; Correlation calculations represent Pearson's coefficient.

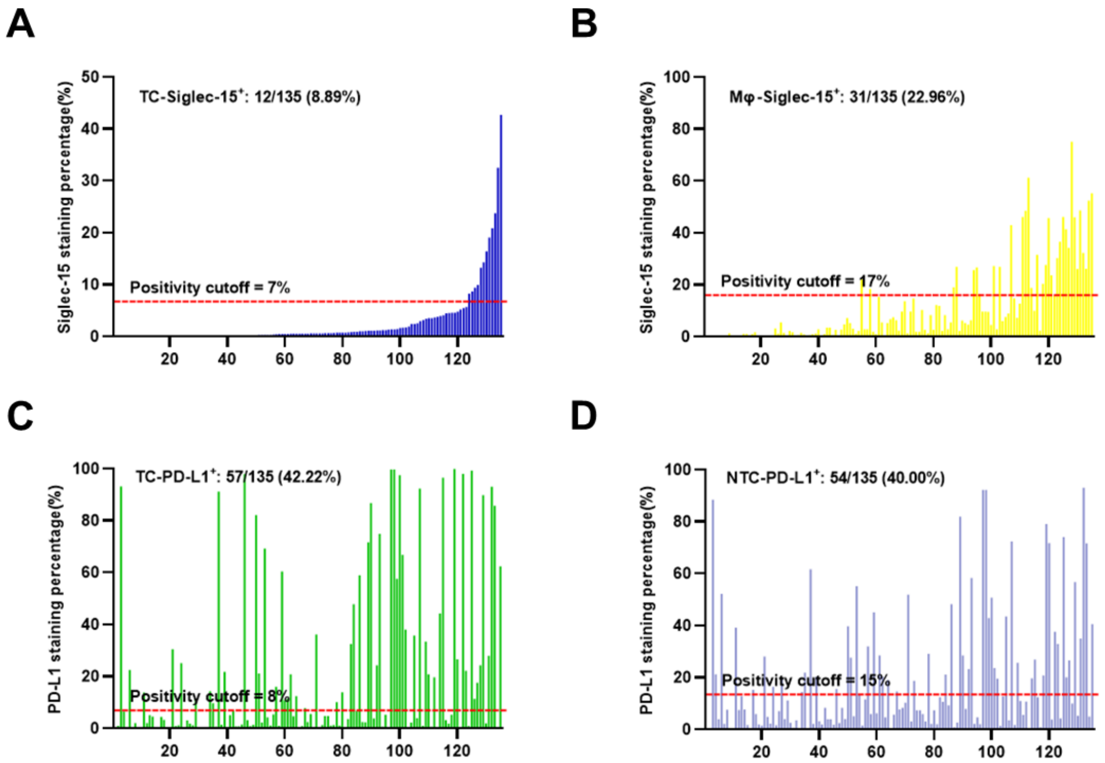

Fig. S2. Staining percentage of Siglec-15 and PD-L1 in NSCLC tissues. (A-D) Distribution of TC-Siglec-15 (A), Mφ-Siglec-15 (B), TC-PD-L1 (C) and NTC-PD-L1 (D) staining percentage in the primary NSCLC cohort. Numbers and percentage of patient cases with positive Siglec-15 staining are shown.

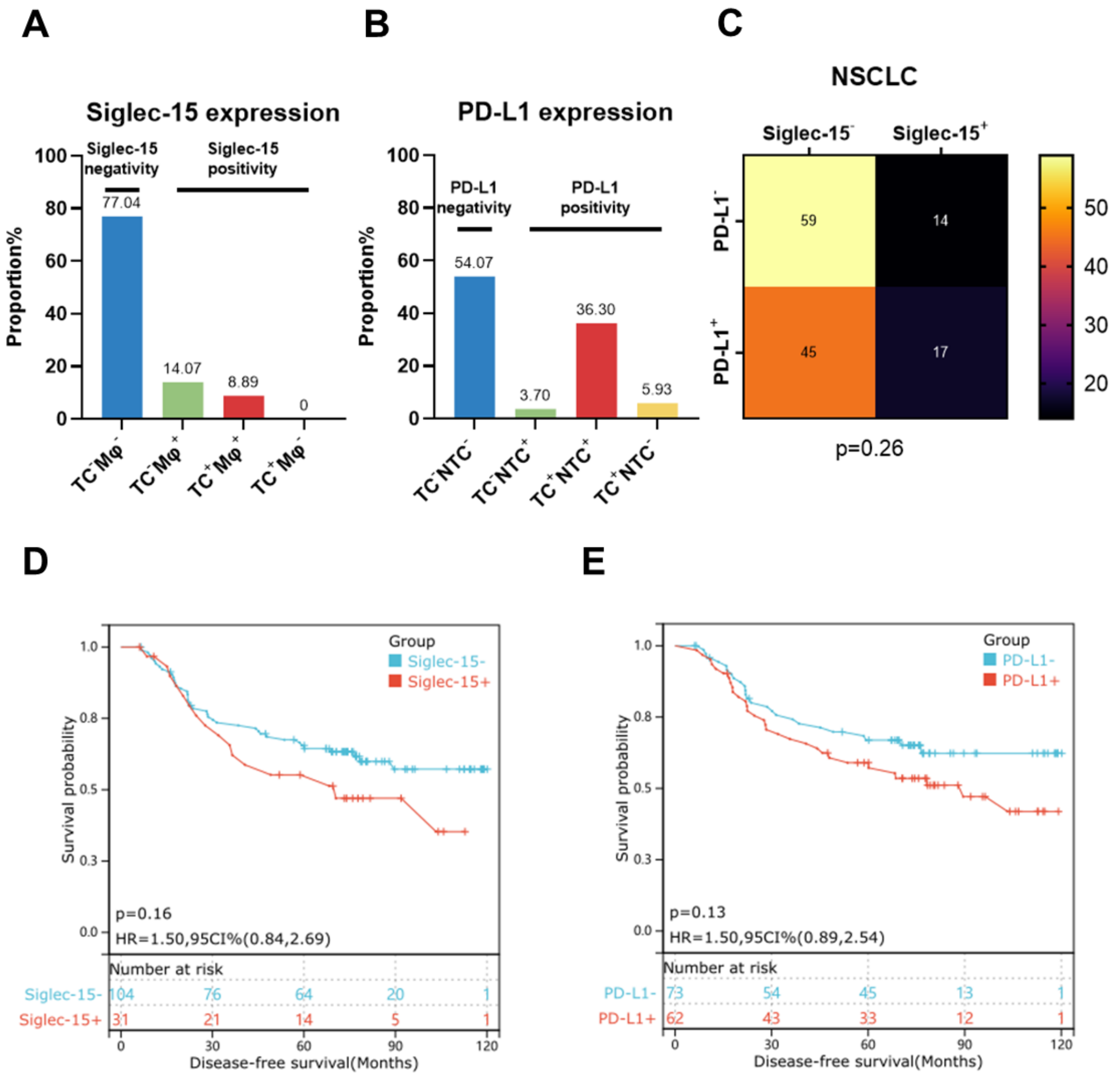

Fig. S3. Different expression patterns of Siglec-15 and PD-L1 in NSCLC. (A) NSCLC samples were divided into 4 groups according to their Siglec-15 expression patterns. (B) NSCLC samples were divided into 4 groups according to their PD-L1 expression patterns. (C) Chi-Square test showed the relationship between Siglec-15 and PD-L1. (D) Comparison of disease-free survival between patients with Siglec-15 positivity and negativity. (E) Comparison of disease-free survival between patients with PD-L1 positivity and negativity.

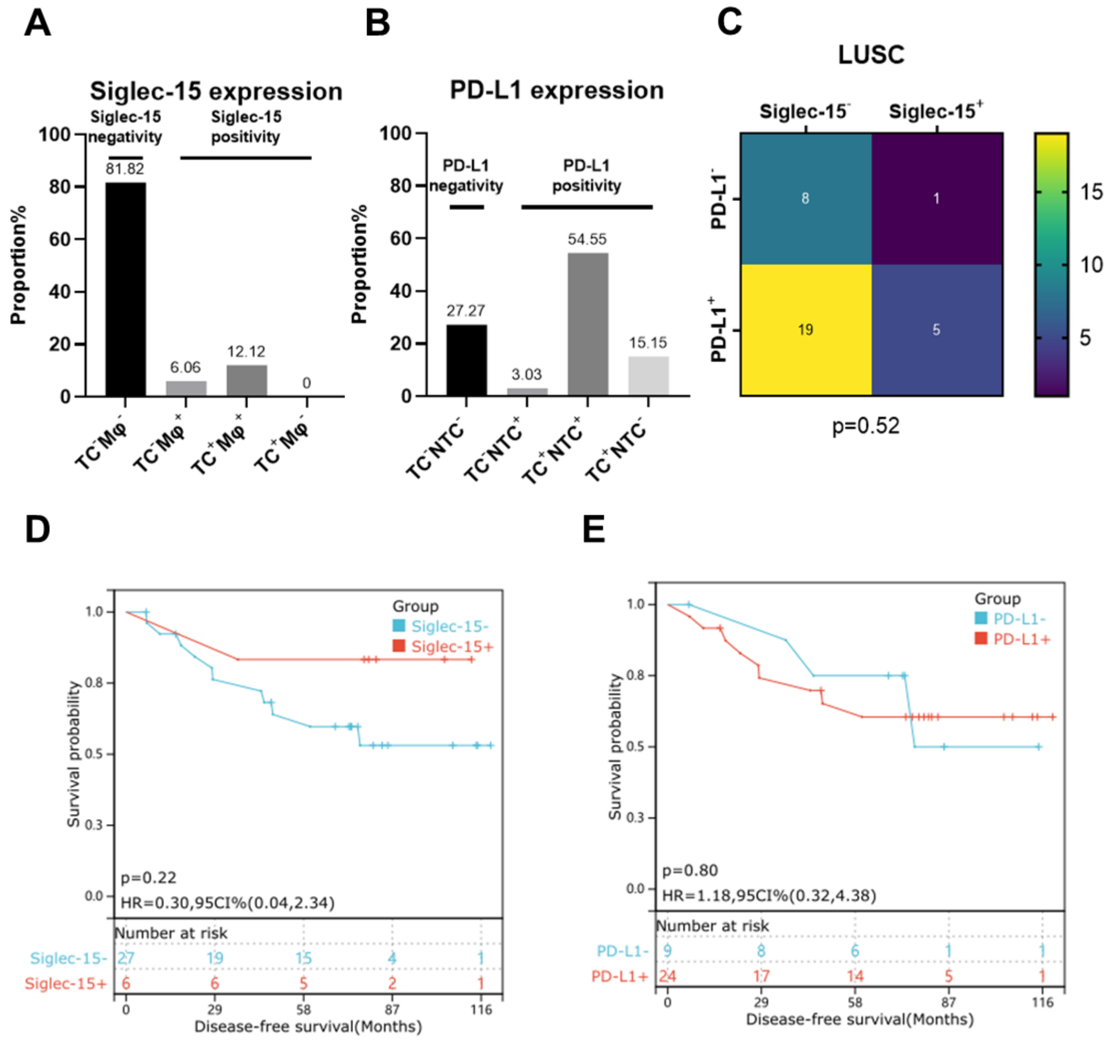

Fig. S4. Different expression patterns of Siglec-15 and PD-L1 in LUSC. (A) LUSC samples were divided into 4 groups according to their Siglec-15 expression patterns. (B) LUSC samples were divided into 4 groups according to their PD-L1 expression patterns. (C) Chi-Square test showed the relationship between Siglec-15 and PD-L1. (D) Comparison of disease-free survival between patients with Siglec-15 positivity and negativity. (E) Comparison of disease-free survival between patients with PD-L1 positivity and negativity.

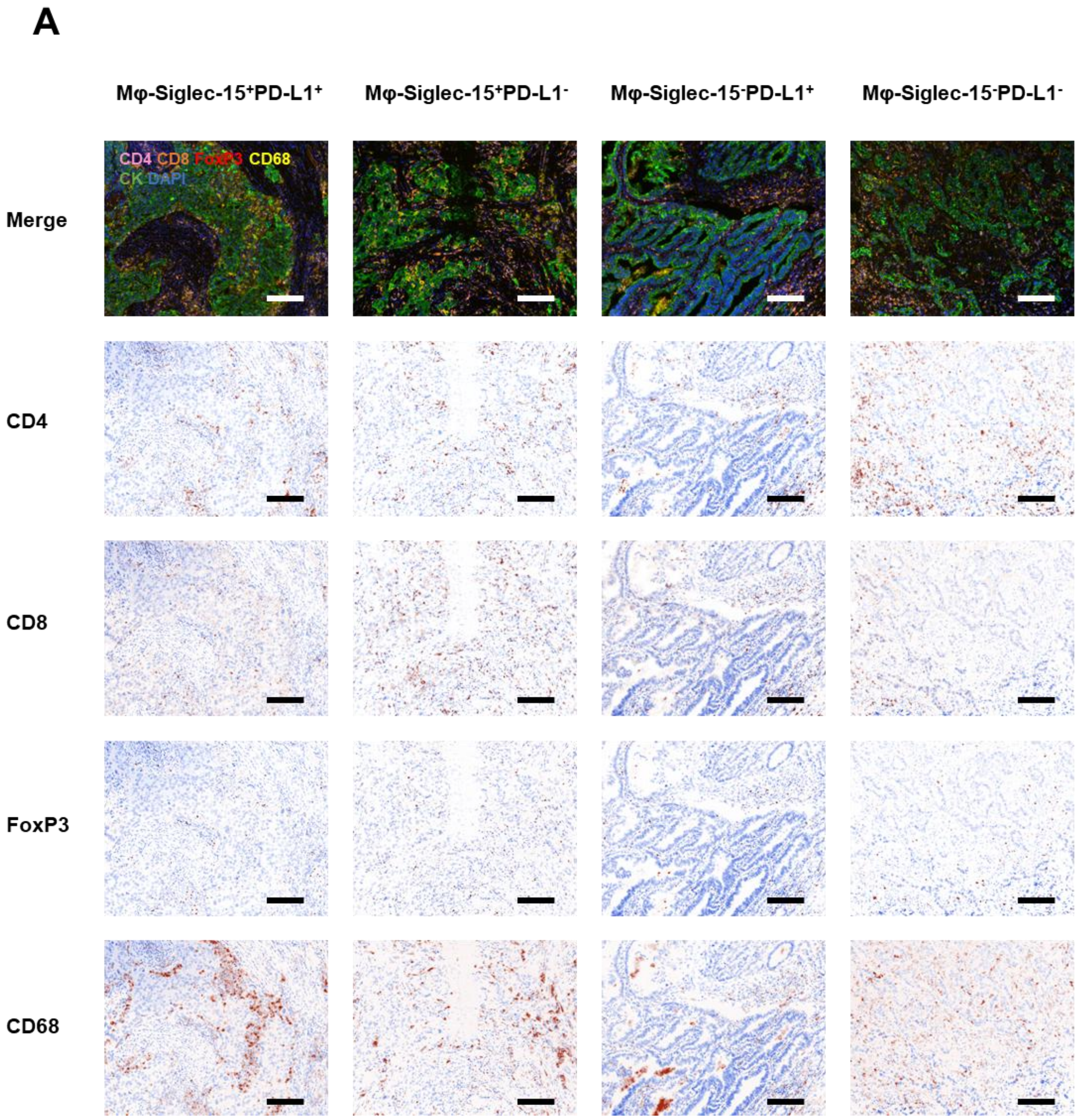

Fig. S5. Representative images of LUAD tissue section with the indicated markers. Scale bar, 50μm.

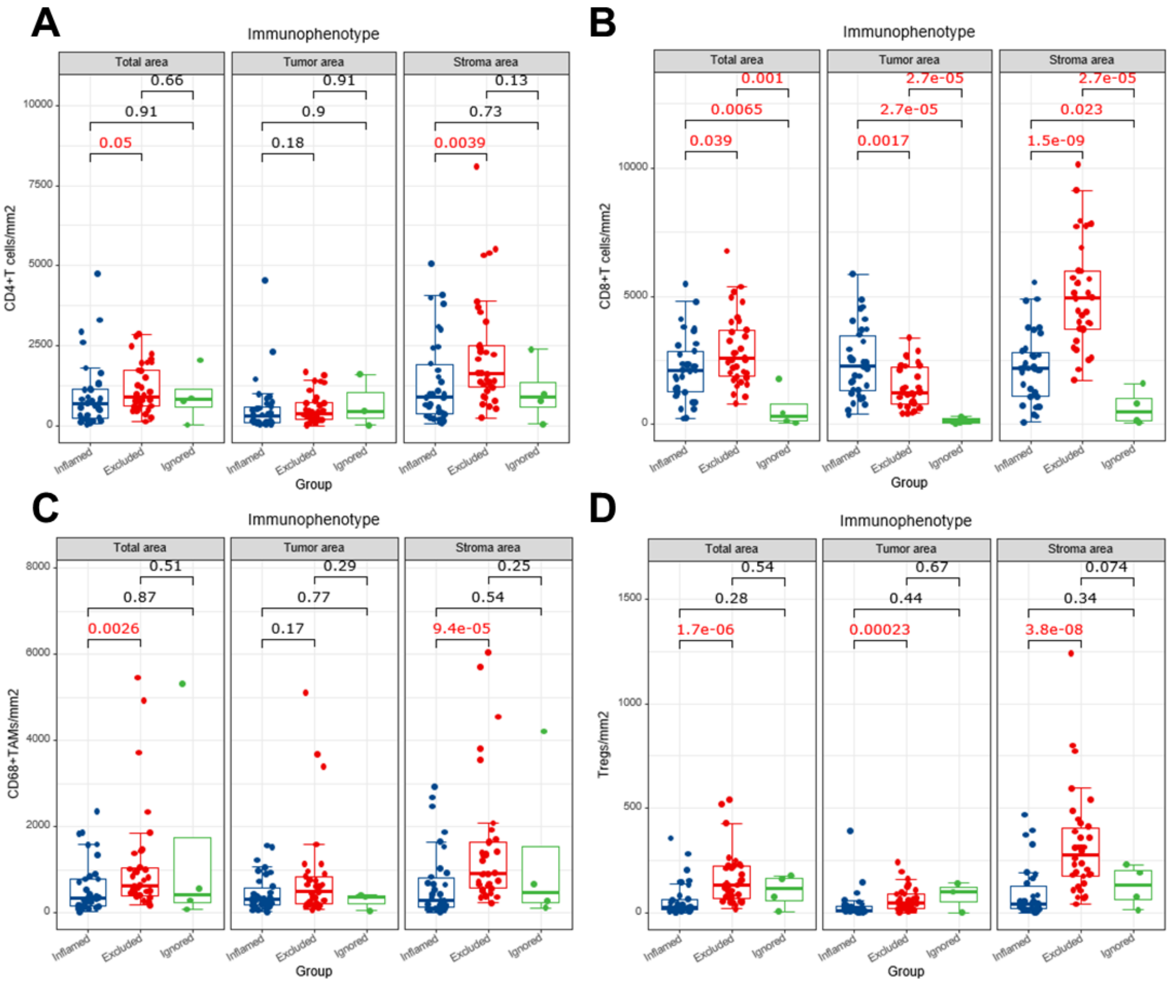

Fig. S6. Density of immune cells among the immunophenotypes.

Among the immunophenotypes in non-metastasis LUAD patients, box plot showed the density of (A) CD4$^+$T cells, (B) CD8$^+$T cells, (C) CD68$^+$TAMs and (D) CD4$^+$FoxP3$^+$Tregs. The bar indicates mean with 95% CI.

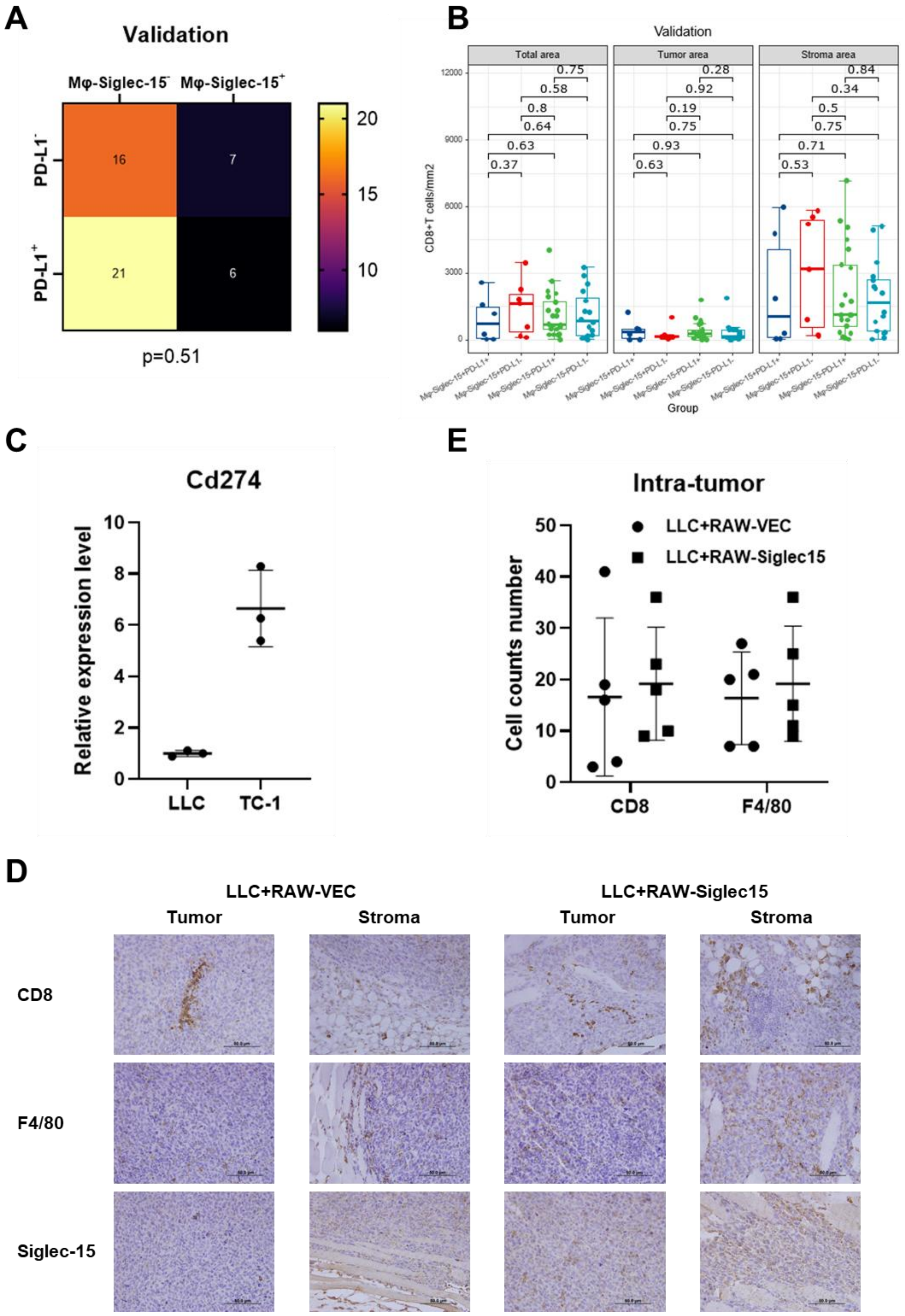

Fig. S7. Validation of the immunophenotypes.

(A) Chi-Square test showed the relationship between Mφ-Siglec-15 and PD-L1 in the validation cohort. (B) The density of CD8^{+}T cells among Mφ-Siglec-15 and PD-L1 patterns in the validation cohort. The bar indicates mean with 95% CI. (C) The

expression of Cd274 in LLC and TC-1 detected by qPCR (n = 3 per group). The bar indicates mean with SD. (D) Representative slide images of immune cells and Siglec-15. Scale bar, 50μm. (E) Comparison of number of intra-tumor immune cells between two groups in vivo (n = 5 per group). The bar indicates mean with SD.

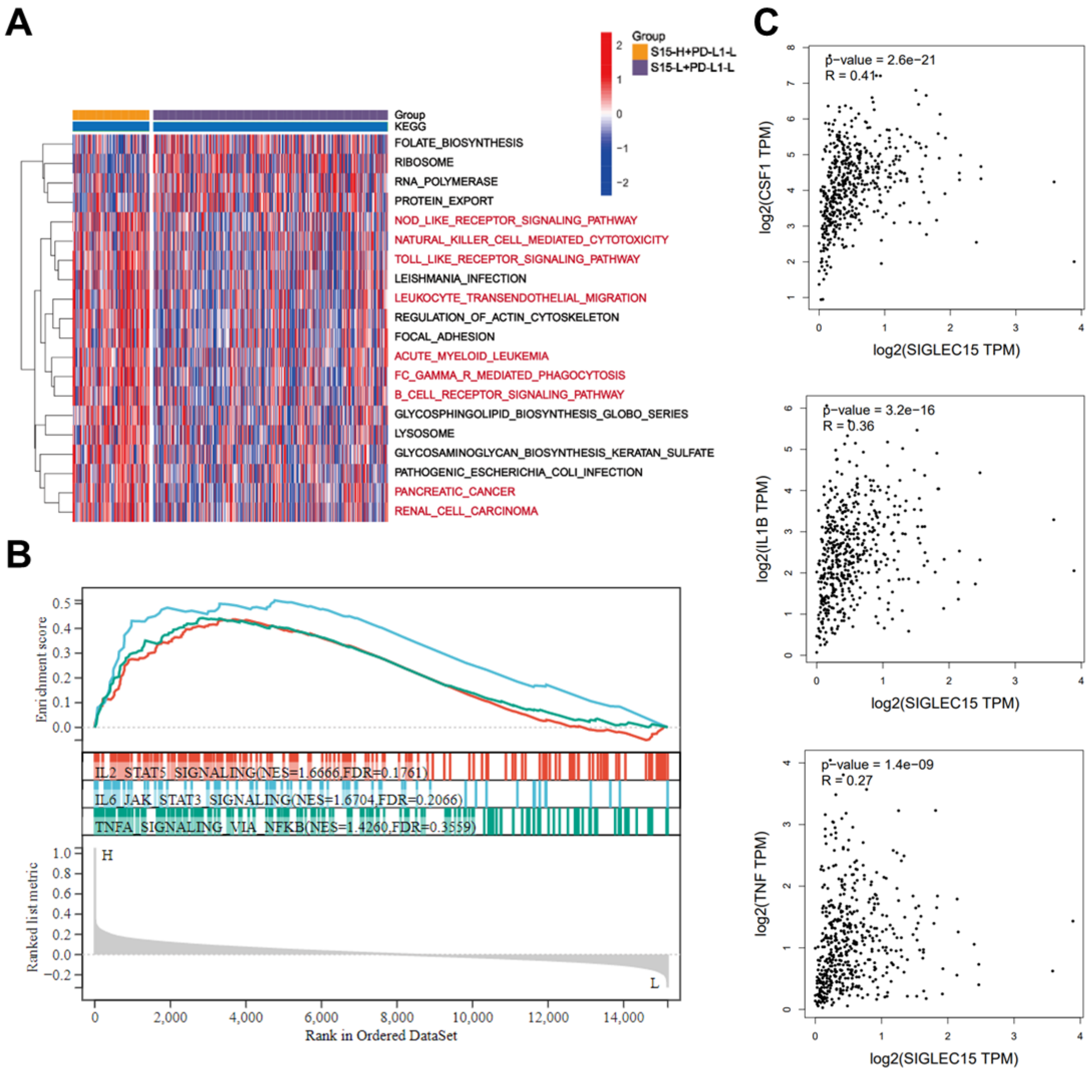

Fig. S8. Pathway analysis of Siglec-15 in LUAD from TCGA.

(A) Heatmap of KEGG pathway enrichment analysis between Siglec-15-H+PD-L1-L and Siglec-15-L+PD-L1-L showed the immune-related terms in red. NES ≥ 1.0, FDR < 0.25. (B) Representative terms of Hallmark enrichment analysis between Siglec-15-H+PD-L1-L and Siglec-15-L+PD-L1-L. (C) The Spearman correlation analysis between Siglec-15 and cytokines.
